# Tumor exosome-mediated promotion of adhesion to mesothelial cells in gastric cancer cells

**DOI:** 10.18632/oncotarget.10869

**Published:** 2016-07-28

**Authors:** Tomohiro Arita, Daisuke Ichikawa, Hirotaka Konishi, Shuhei Komatsu, Atsushi Shiozaki, Shinpei Ogino, Yuji Fujita, Hidekazu Hiramoto, Junichi Hamada, Katsutoshi Shoda, Toshiyuki Kosuga, Hitoshi Fujiwara, Kazuma Okamoto, Eigo Otsuji

**Affiliations:** ^1^ Division of Digestive Surgery, Department of Surgery, Kyoto Prefectural University of Medicine, Kyoto, Japan

**Keywords:** gastric cancer, exosome, peritoneal metastasis, fibronectin 1, laminin gamma 1

## Abstract

**Background:**

Peritoneal metastasis consists of a highly complex series of steps, and the details of the underlying molecular mechanism remain largely unclear. In this study, the effects of tumor-derived exosomes (TEX) on the progression of gastric cancers were investigated in peritoneal metastasis.

**Results:**

TEX were internalized in both mesothelial and gastric cancer cells in a cellular origin non-specific manner. Internalization of TEX into mesothelial cells promoted significant adhesion between mesothelial and gastric cancer cells, and TEX internalization into gastric cancer cells significantly promoted migratory ability, while internalization of mesothelial cell-derived exosomes did not. Expression of adhesion-related molecules, such as fibronectin 1 (FN1) and laminin gamma 1 (LAMC1), were increased in mesothelial cells after internalization of TEX from gastric cancer cell line and malignant pleural effusion.

**Methods:**

TEX were extracted from cell-conditioned medium by ultracentrifugation. The effects of TEX on the malignant potential of gastric cancer were investigated in adhesion, invasion, and proliferation assays. PCR array as well as western blotting were performed to determine the underlying molecular mechanisms. The molecular changes in mesothelial cell after internalization of TEX derived from malignant pleural effusion were also confirmed.

**Conclusions:**

TEX may play a critical role in the development of peritoneal metastasis of gastric cancer, which may be partially due to inducing increased expression of adhesion molecules in mesothelial cells.

## INTRODUCTION

Peritoneal metastasis is one of the most common patterns of recurrence in gastric cancer patients [1]. Formation of peritoneal metastasis consists of a highly complex series of mechanisms, and the details of these steps remain largely unknown. However, various factors, including tumor and host factors, have been recognized as playing some roles in metastasis formation. Serosal involvement by the primary tumor and subsequent intraperitoneal release of cancer cells are crucially important factors for metastatic formation. However, presence of intraperitoneal free cancer cells does not necessarily indicate peritoneal dissemination [2], and several other factors, such as adhesion factors of the cancer cells and the host immune system, are also involved in the peritoneal metastatic process [3-6].

Accumulating evidence indicates that exosomes play important roles for intercellular communication [7-12]. Exosomes are small membrane vesicles measuring 50-100 nm in diameter and are secreted from various cell types, with tumor cells secreting excessive amounts of exosomes compared with non-tumor normal cells. Exosomes derived from cancer cells carry mRNA, microRNA, and proteins that can communicate signals to local and remote cells and tissues. In melanoma cells, Hood et al. reported that exosomes released by cancer cells induce an environment suitable for lymph node metastasis [14].

Based on these findings, we hypothesized whether tumor-derived exosomes (TEX) may facilitate peritoneal dissemination in gastric cancer. We investigated the possible involvement of TEX on the development of peritoneal dissemination by analyzing the effects of TEX on the adhesive and invasive abilities of tumor and mesothelial cells. Following this, we also investigated the molecular mechanisms underlying the development of peritoneal metastasis induced by internalization of TEX in gastric cancer.

## RESULTS

### Purification of exosomes and internalization

**Figure 1:**
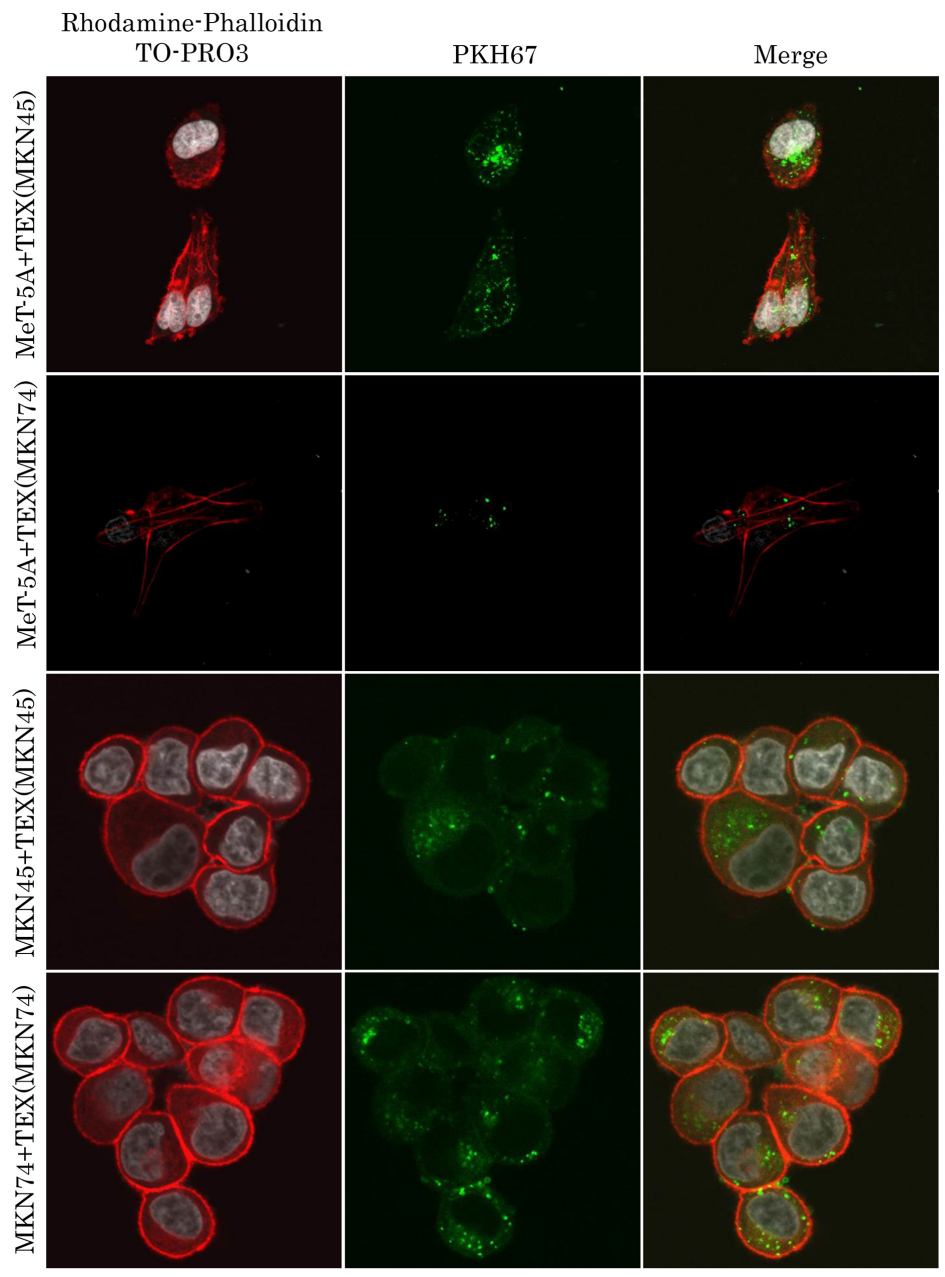
TEX internalization into mesothelial and gastric cancer cells Representative images of immunofluorescence microscopy of exosomes (green) co-cultured with mesothelial and gastric cancer cells.

TEX were well internalized into both mesothelial and gastric cancer cells in a cellular origin non-specific manner (Figure 1). Isolation of exosomes was confirmed by Western blotting with exosomal markers CD9 and CD63 (data not shown). Internalized TEX were detected in the cellular cytoplasm without morphological change. Exosomes from mesothelial cells were also observed internalized into both mesothelial and gastric cancer cells (Supplementary Figure S1).

### Influence of TEX on the malignant phenotype of gastric cancer cells

**Figure 2:**
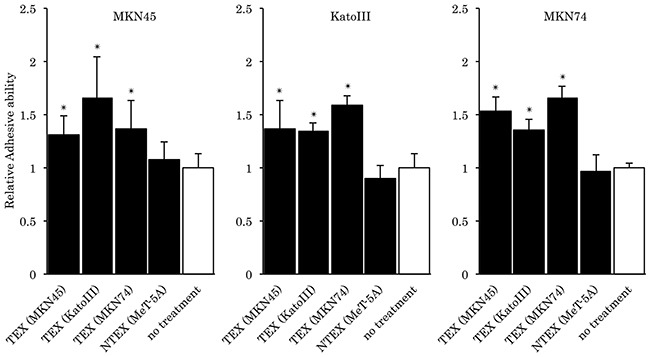
Relative fluorescence in the adhesion assay Each assay was performed ten times and normalized to a no-treatment series. *p<0.05 compared to no-treatment series. NTEX; non-tumor derived exosome.

To analyze whether the adhesive ability of gastric cancer cells to mesothelial cells was affected by the presence of TEX, an adhesion assay was performed using TEX-internalized MeT-5A cells. TEX significantly promoted the adhesive ability of gastric cancer cells to mesothelial cells in a cellular origin non-specific manner, while exosomes from mesothelial cells did not induce such an effect (Figure 2).

**Figure 3:**
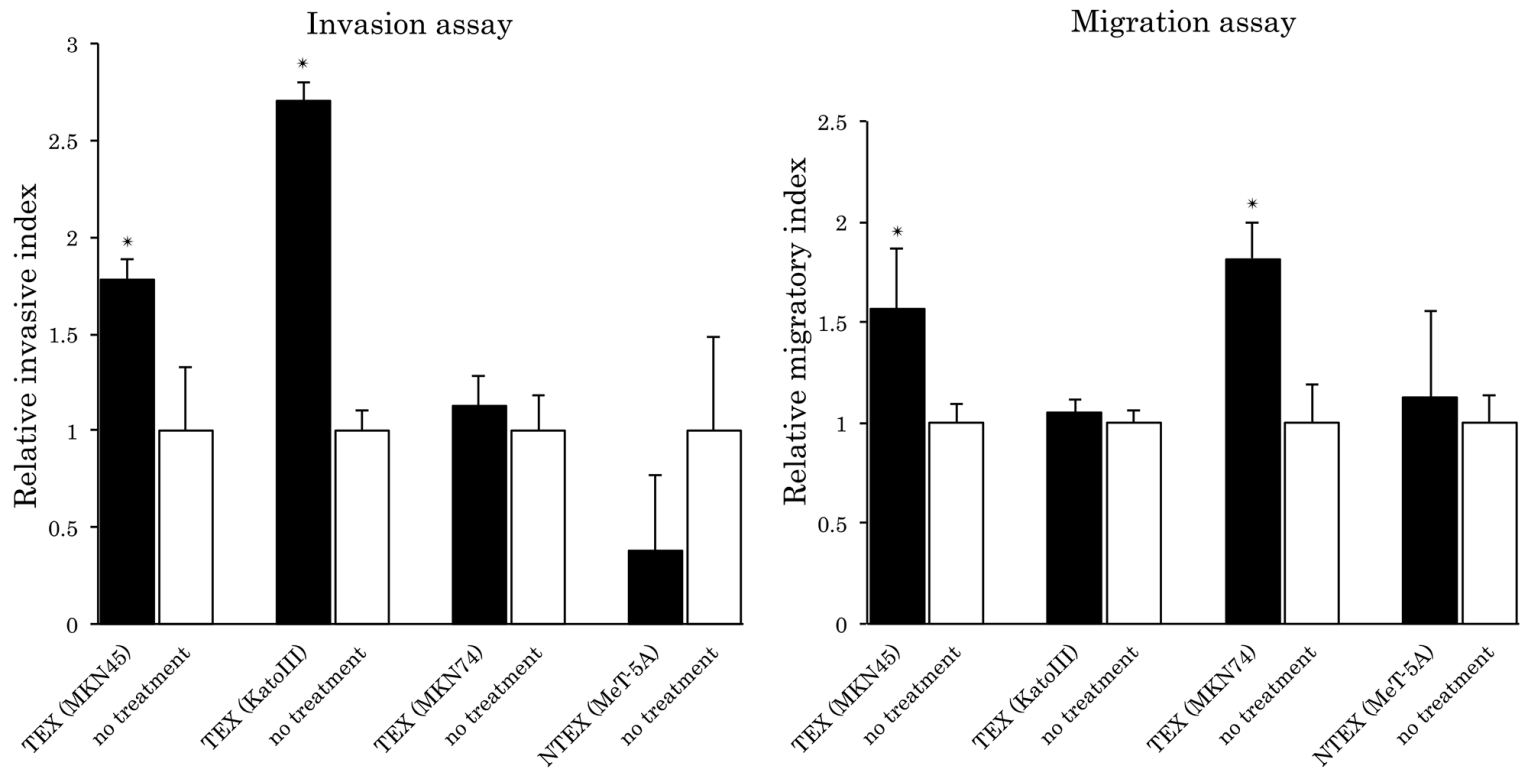
Relative invasive and migratory index Each assay was normalized to a no-treatment series. *p<0.05 compared to no-treatment series.

Invasion and migration assays were also performed using TEX-internalized gastric cancer cells. TEX purified from MKN45 and KatoIII cells significantly enhanced the invasive ability of MKN45 cells, and TEX purified from MKN45 and MKN74 enhanced MKN45 cell migration. The impact and acquisition of malignancy of the recipient cells differed depending on the TEX origin. The enhancement of invasive and migratory abilities was not observed by addition of exosomes from mesothelial cells (Figure 3).

### Effect of TEX internalization on molecular mechanisms

**Figure 4:**
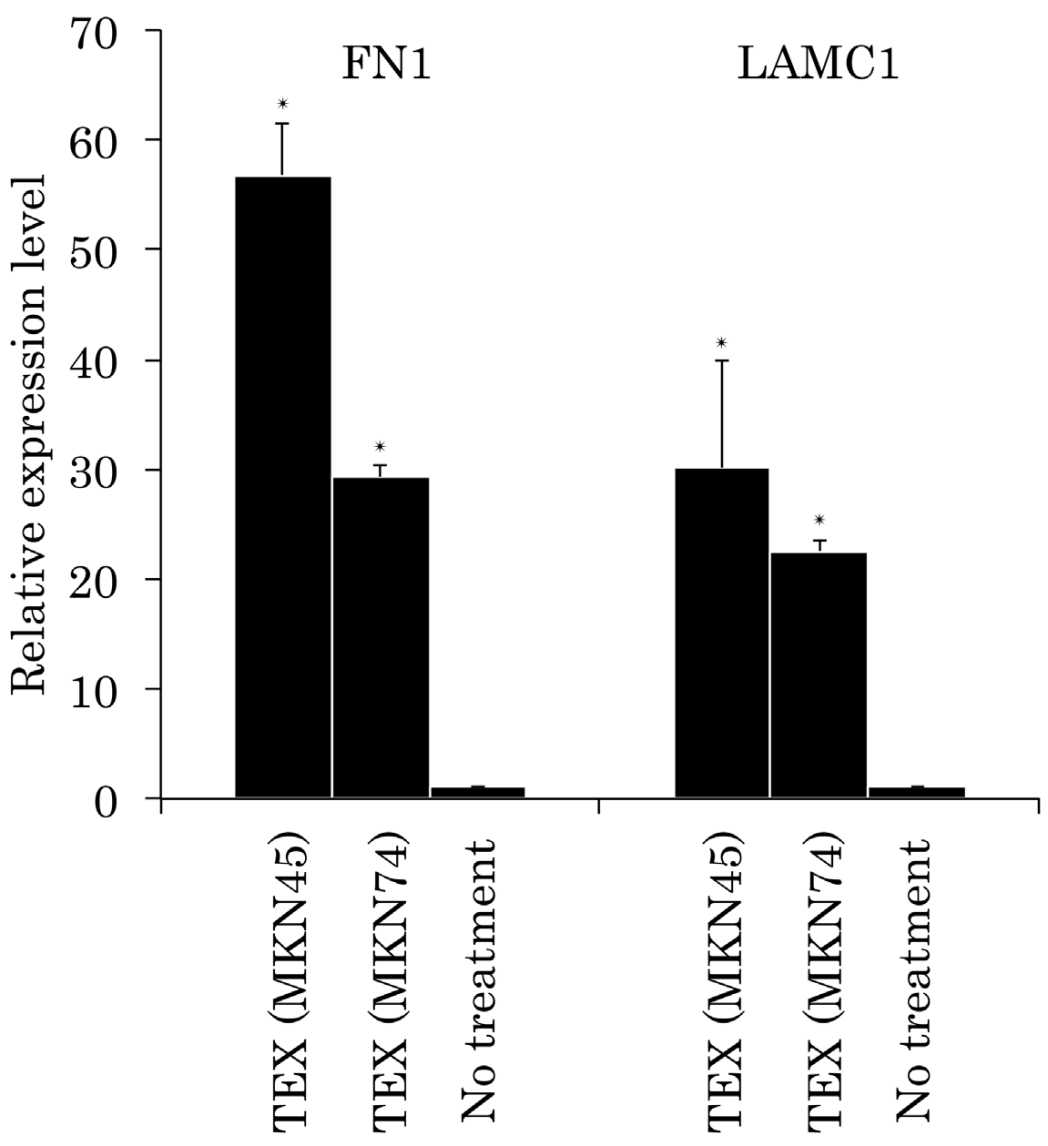
Relative expression levels of FN1 and LAMC1 in TEX-internalized Met-5A cells by qRT-PCR analyses The levels of FN1 and LAMC1 were calculated using the ΔΔCt method relative to ACTB. *p<0.05 compared to no-treatment series.

**Figure 5:**
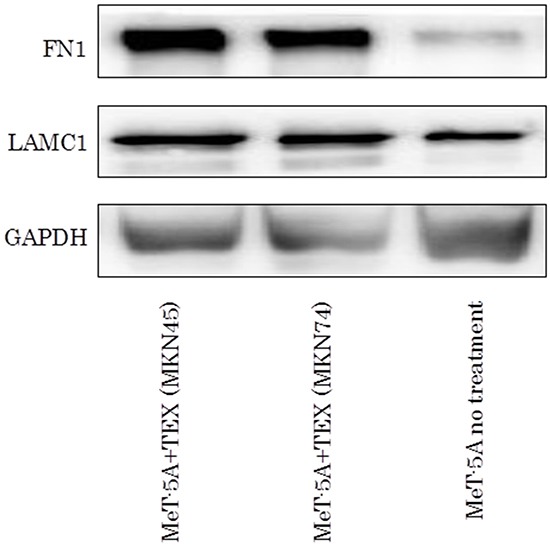
The western blotting assay of FN1 and LAMC1 in TEX-internalized MeT-5A cells.

Following the cellular function assays, gene alterations induced by internalization of TEX were analyzed using PCR array of the recipient cells. We used a PCR array kit with extracellular matrix and adhesion molecule targets to clarify the molecular mechanism underlying the enhancement of adhesive ability of gastric cancer cells to mesothelial cells. Both FN1 and LAMC1 expression were upregulated in MeT-5A cells treated with TEX from either MKN45 or MKN74 cells (Supplementary Figure S2). Significant increases in expression of the two genes were confirmed by real time qRT-PCR (Figure 4). Protein expression of these molecules was also examined by Western blotting, showing that TEX significantly increased expression of FN1 and LAMC1 in MeT-5A cells (Figure 5).

PCR array with metastasis-involved target genes was also performed to clarify the molecular mechanism underlying the enhancement of the migratory ability of gastric cancer cells. However, we failed to identify any specific molecules due to little overlap in expression changes in the two cell lines assessed.

### Effect of TEX in clinical specimen on FN1 and LAMC1

**Figure 6:**
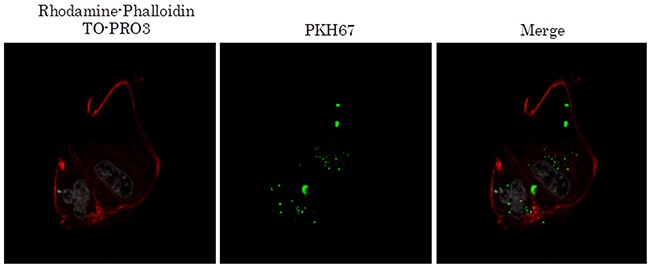
TEX from malignant pleural effuseon internalized into mesothelial cells Representative images of immunofluorescence microscopy of exosomes (green) co-cultured with mesothelial and gastric cancer cells.

**Figure 7:**
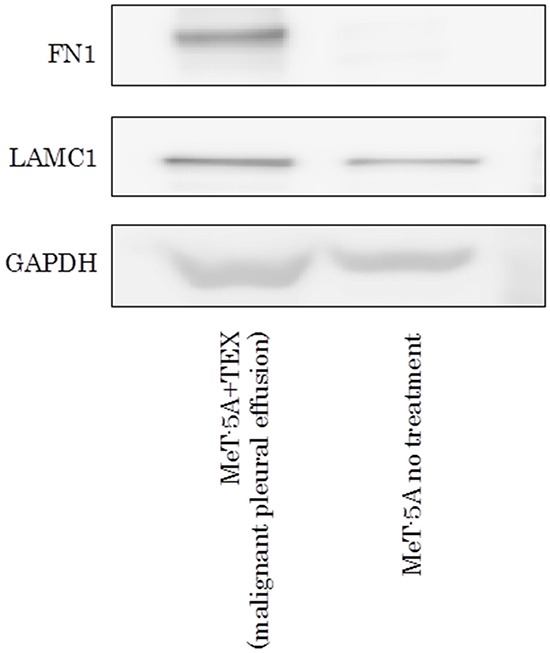
Western blotting assay of FN1 and LAMC1 in MeT-5A ingested TEX from malignant pleural effusion.

TEX from malignant pleural effusion were also internalized in mesothelial cells (Figure 6). Furthermore, protein expression of TEX–internalized mesothelial cells revealed increased expression of FN1 and LAMC1 by Western blotting (Figure 7). The limited amount of pleural effusion precluded further examinations using TEX from pleural effusion.

Subsequently, 10 individual peritoneal tissues with peritoneal dissemination and 5 without dissemination were collected from surgical specimens. Immunohistochemistry demonstrated higher expression of FN1 and LAMC1 in the disseminated peritoneal membrane than in the benign peritoneal membrane (Supplementary Figure S3).

## DISCUSSION

The mechanism governing formation of peritoneal metastasis remains to be fully clarified; however, cancer cells are thought to undergo a series of sequential steps for formation of peritoneal dissemination, as follows: 1) visceral serosal involvement of tumor tissues; 2) exfoliation of cancer cells from the primary tumor; 3) adhesion of the free cancer cells to the peritoneal mesothelial cells; 4) invasion of the cancer cells through the peritoneal membrane and formation of peritoneal metastasis. Recent studies have demonstrated that various molecules are involved in peritoneal dissemination. For instance, protease activity is enhanced by MMPs, leading to increased cell motility within the surrounding tissue, ECM, and stromal cells [3]. The integrity and strength of cell-cell contacts are also decreased by the loss of E-cadherin in cancer cells [3, 4]. CD44 functions as a ligand-binding receptor by interacting with the ECM and other extracellular components [5]. Increased expression of integrin is responsible for increased adhesion of cancer and mesothelial cells [6].

Exosomes are vesicles ranging from 40 to 1,000 nm in size that are released by a variety of cultured cells, and mediate transfer of mRNA, microRNA, and proteins from cell to cell [11, 12]. In malignant cancers, TEX induce vascular permeability and promote metastasis [13]. Furthermore, TEX have been reported to prepare sentinel lymph nodes for tumor metastasis [14].

In the present study, TEX promoted the adhesive ability of gastric cancer cells and normal mesothelial cells, and also enhanced the invasive and migratory capabilities of gastric cancer cells, although TEX had no effect on tumor cell proliferation (data not shown). These phenomena suggest that TEX may be involved in inter-cellular communication and condition an advantageous microenvironment for peritoneal dissemination via alteration of recipient cells, including cancer and normal mesothelial cells.

According to the current version of the exosome content database, Exocarta, 8,815 proteins, 2,375 mRNAs, 764 microRNAs, and 194 lipids have been identified in the exosomes of many different cell types and from multiple organs (http://www.exocarta.org). We attempted to identify the molecules in the TEX or TEX-internalized recipient cells that mediated the formation of the advantageous microenvironment for peritoneal metastasis. PCR array of ECM and adhesion-related gene targets suggested that FN1 and LAMC1 may play key roles in cell adhesion. These molecules were confirmed by qRT-PCR to be upregulated in mesothelial cells treated with TEX. Sugiyama and colleagues have previously reported that exosomal microRNAs regulate FN1 expression in mesothelial cells [15]. FN1 and LAMC1 ligands include integrin, a regulator of cell adhesion and a prognostic factor of gastric cancer [16-18].

We were unable to identify specific TEX-induced gene expression alterations by PCR array of metastasis-related gene targets; changes in expression at 24 and 48 hours after TEX internalization completely differed from one another and precluded analysis. Furthermore, the effect of TEX internalization on the microRNA expression profile of recipient cells also resulted in convoluted microarray results (data not shown). We are now further investigating the mechanism of TEX-mediated conditioning of the microenvironment for peritoneal dissemination by assessing TEX microRNA and proteins. Hoshino and colleagues recently demonstrated that exosomal integrins determine organotropism and interact with the cell-associated extracellular matrix such as fibronectin and laminin, mediating exosomal uptake in specific target organs [19].

Exosomes are known to contain proteins, mRNA, and microRNA from the cytoplasm of the donor cell, and these may function in the recipient cell and trigger dynamic transformation leading to a cancer-preferable microenvironment. Utilizing microvesicles, several researchers are currently pursuing their possible application in a drug delivery system; neuronal exosomes have been shown to accelerate amyloid-β formation, which may offer a possible target for therapy of Alzheimer disease [20].

In conclusion, intra-peritoneal TEX, secreted from gastric cancer cells, are internalized into both the cancer cell itself and mesothelial cells to promote peritoneal dissemination. The present results warrant further studies to fully elucidate the role of TEX in peritoneal dissemination.

## MATERIALS AND METHODS

### Cell culture

The human gastric cancer cell lines KatoIII (RCB2088), MKN45 (RCB1001), and MKN74 (RCB1002) were purchased from RIKEN Bio Resource Center (Tokyo, Japan), and the human normal mesothelial cell line MeT-5A (CRL-9444) was purchased from ATCC (Manassas, VA, USA). These cell lines were maintained in RPMI medium (Nacalai Tesque, Kyoto, Japan) supplemented with 10% exosome-depleted fetal bovine serum (System Biosciences, CA, USA), 100 U/mL penicillin and 100 μg/mL streptomycin. The flasks were kept in a humidified incubator at 37°C with 5.0% CO_2_.

### Exosome preparation

Cell culture medium was collected after 3 days incubation with exosome-donor cells, and the number of cells was counted simultaneously. Medium was filtered through a 0.22 μm filter (Merck Millipore, Darmstadt, Germany) and ultracentrifuged at 100,000 g for 70 minutes at 4°C. The pellet was washed with PBS and ultracentrifuged again. In all assays, TEX purified from media conditioned by 30-fold the number of recipient cells were used.

Malignant pleural effusion was aspirated from a 74-year-old man with advanced gastric cancer for alleviation of symptoms. The pleural effusion was diagnosed as class V in cytological examination. With informed consent, 100 mL of excess effusion was used for TEX extraction. TEX were purified in the same manner as cell culture medium.

### Immunofluorescence staining

TEX were ultracentrifuged at 100,000 g for 70 minutes at 4°C after incubation with PKH67 green (Sigma-Aldrich, MO, USA) for 30 minutes at room temperature. MeT-5A cells were seeded on a cell culture slide (SPL Life Sciences, Korea) for 12 hour prior to PKH67-labeled TEX addition. After washing five times with PBS gently, 4% paraformaldehyde was added to the cells. Nuclei were visualized by TO-PRO3 (Invitrogen-Molecular Probes) and the actin cytoskeleton by rhodamine-phalloidin (Setareh Biotech). Images were acquired by confocal laser scanning microscopy (FV1000, Olympus, Tokyo, Japan).

### Adhesion assay

Adhesion assays using the Endothelial Cell Adhesion Assay Kit (Chemicon International, Temecula, CA, USA; Cat. No. ECM645) were performed following the manufacturer's instructions, with mesothelial cells (MeT-5A) instead of endothelial cells. Briefly, 4.0×10^5^ MeT-5A cells in each well were cultured in RPMI for 48 hours with TEX derived from 1.2×10^6^ MKN45 or MKN74. After the MeT-5A cells had been treated with tumor necrosis factor-α, 1.0×10^5^ Calcein AM^®^-labeled gastric cancer cells were seeded in each well and incubated for 30 min. After gentle and complete removal of the supernatant, including the floating cancer cells, the fluorescent signal was read with a fluorescence plate reader using a 485/560 nm excitation/ emission filter set. Each assay series was performed ten times, and fluorescence was normalized to a no-treatment series.

### Invasion and migration assay

Invasion and migration assays were performed six times using the BD BioCoat Matrigel^TM^ Invasion Chamber kit (BD Biosciences, NJ, USA) following the manufacturer's protocols. In brief, 1×10^5^ gastric cancer cells (MKN45) were loaded in the upper Boyden chamber in RPMI supplemented with 10% exosome-depleted fetal bovine serum, 100 U/mL penicillin, 100 μg/mL streptomycin, and TEX. The lower chamber contained RPMI without FBS. After incubation for 48 hours at 37°C, duplicate membranes were processed and evaluated by counting cells in 10 random fields under a microscope. The migration assay was performed in parallel with the invasion assay under the same conditions, except using an uncoated membrane.

### PCR array analysis

The Human Extracellular Matrix and Adhesion Molecules RT^2^ PCR array (Cat. No. PAHS-013Z) and Human Tumor Metastasis RT^2^ PCR array (Cat. No. PAHS-028Z) were purchased from QIAGEN (Hamburg, Germany). Total RNA was extracted from 1×10^6^ MeT-5A, MKN45, and MKN74 cells incubated with TEX derived from 3×10^7^ MKN45 or MKN74 at 37°C for 48 hours using the miRNeasy Mini kit (QIAGEN, Hamburg, Germany) following the manufacturer's protocols. Reverse transcription was performed using the RT^2^ First Strand kit (QIAGEN, Hamburg, Germany) according to the manufacturer's protocols. The RT^2^ PCR array was performed using the Step One Plus Real-time PCR system (Applied Biosystems) and analyzed by a web-based analysis program (http://www.qiagen.com).

### Real time qRT-PCR validation

Total RNA of TEX-incubated cells was extracted using the miRNeasy Mini kit (QIAGEN, Hamburg, Germany) following the manufacturer's instructions. The reverse transcription reaction was carried out using the High Capacity cDNA RT kit (Applied Biosystems, Foster City, CA, USA). The expression levels of fibronectin 1 (FN1) and laminin gamma 1 (LAMC1) were quantified in duplicate by quantitative real time-polymerase chain reaction (qRT-PCR) using the human TaqMan Gene Expression Assay Kit (Applied Biosystems) following the manufacturer's protocols. In brief, quantitative PCR analyses were performed using the Step One Plus Real-time PCR system (Applied Biosystems), and cycle threshold (Ct) values were calculated with the Step One Plus Software version 2.2.2 (Applied Biosystems). The levels of FN1 and LAMC1 were calculated using the ΔΔCt method relative to actin β (ACTB). The change in gene expression was expressed with the equation 2^-ΔΔCt^.

### Western blotting

The expression levels of fibronectin 1 (FN1) and laminin, gamma 1 (LAMC1) in MeT-5A with/without TEX incubation were investigated by western blotting. The antibodies for FN1 (Cat. No. HPA027066) and LAMC1 (Cat. No. HPA001909) were purchased from Sigma Life Science (MO, USA). The antibody for glyceraldehyde-3-phosphate dehydrogenase (GAPDH) was from Santa Cruz Biotechnology (CA, USA). The cells were harvested in M-PER lysis buffer (Pierce, Rockford, IL, USA) supplemented with protease inhibitors (Pierce, Rockford, IL, USA). Protein concentration was measured by a modified Bradford assay (Bio-Rad, Hercules, CA, USA). Cell lysates containing 20 μg of total protein were separated by SDS-PAGE and then transferred onto PVDF membranes (GE Healthcare, Piscataway, NJ, USA). The membranes were then probed with the indicated antibodies, and proteins were detected by an ECL Plus Western Blotting Detection System (GE Healthcare, Piscataway, NJ, USA).

### Immunohistochemistry staining

Ten individual peritoneal tissues with peritoneal dissemination and five without dissemination were collected from surgical specimens. These specimens were fixed with 10% formaldehyde in PBS, embedded in paraffin, sectioned into 5-μm thick slices, and subjected to immunohistochemical staining for FN1 and LAMC1 proteins with the avidin–biotin–peroxidase method. In brief, after deparaffinization, endogenous peroxidases were quenched by incubating the sections for 20 min in 3% H_2_O_2_. Antigen retrieval was performed by heating the samples in 10 mmol/L citrate buffer (pH 6.0) at 95°C for 60 min. After treatment with Block Ace (Dainippon Sumitomo Pharmaceutical, Osaka, Japan) for 30 min at room temperature, sections were incubated 4°C overnight with anti-FN1 (1 : 400) and anti-LAMC1 (1 : 750) antibodies. The avidin-biotin-peroxidase complex system (Vectas- tain Elite ABC universal kit; Vector Laboratories Inc., Burlingame, CA, USA) was used for color development with diaminobenzidine tetrahydrochloride. Slides were counterstained with Mayer's hematoxylin.

### Statistics

Statistical analysis was performed using JMP version 10 (ASA Institute, Cary, NC, USA) and SPSS version 20 (IBM Corporation, Armonk, NY, USA). The adhesion assay signal intensity, invasion and migration assay cell counts, and real-time RT-PCR data were all evaluated using the Mann-Whitney U-test. For all analyses, *p* values were considered significant when <0.05.

## SUPPLEMENTARY MATERIALS FIGURES


