# Supplementary material for: Tumor exosome-mediated promotion of adhesion to mesothelial cells in gastric cancer cells
Source: Oncotarget. 2016 Jul 28;7(35):56855–63. doi: 10.18632/oncotarget.10869 (PMC5302957; doi:10.18632/oncotarget.10869)
Supplement: Supplementary file 1 [file oncotarget-07-56855-s001.pdf]

Tumor exosome-mediated promotion of adhesion to mesothelial cells in gastric cancer cells

SUPPLEMENTARY FIGURES

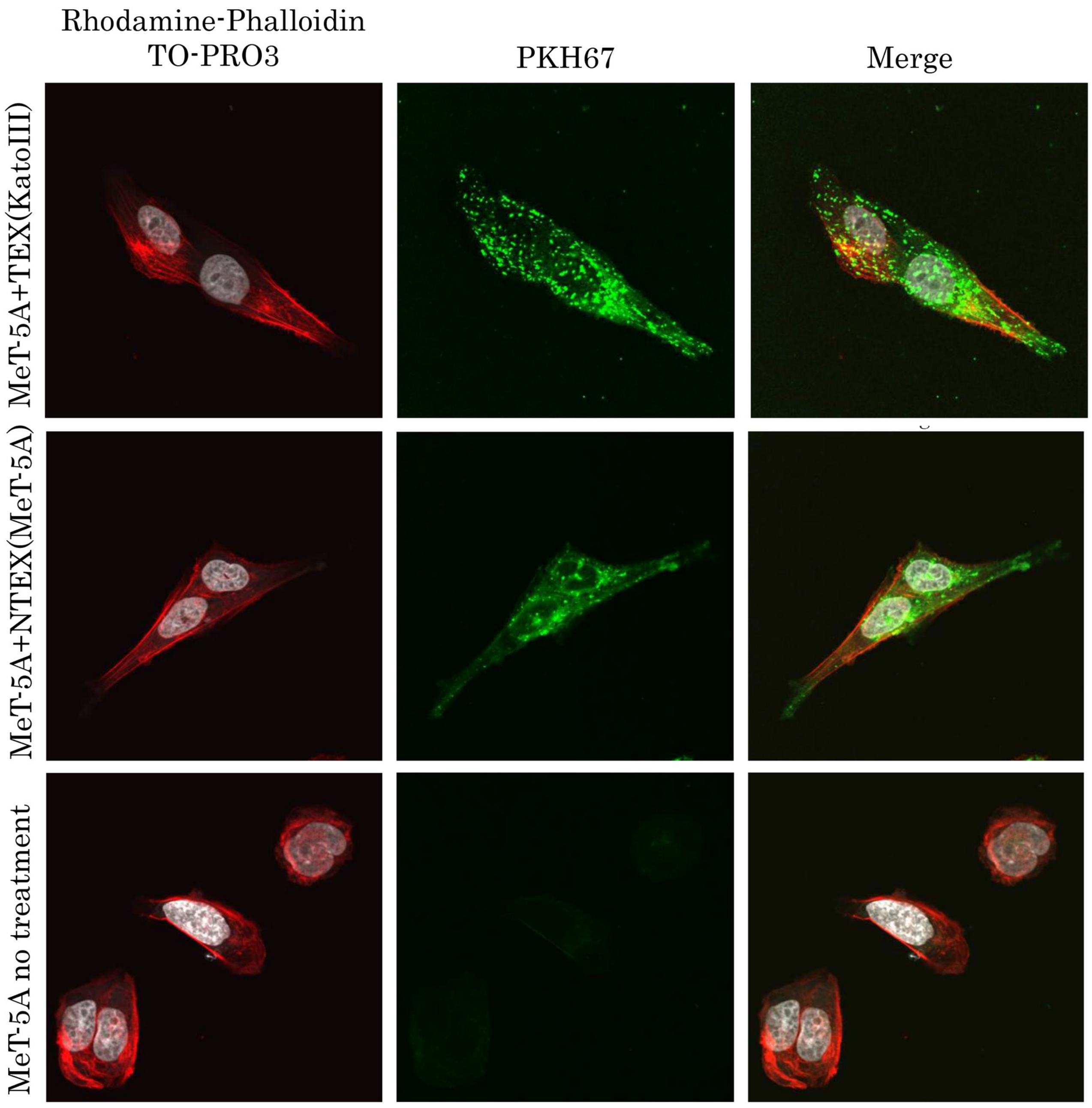

Supplementary Figure S1: TEX from KatoIII and NTEX from MeT-5A internalization into MeT-5A cells.

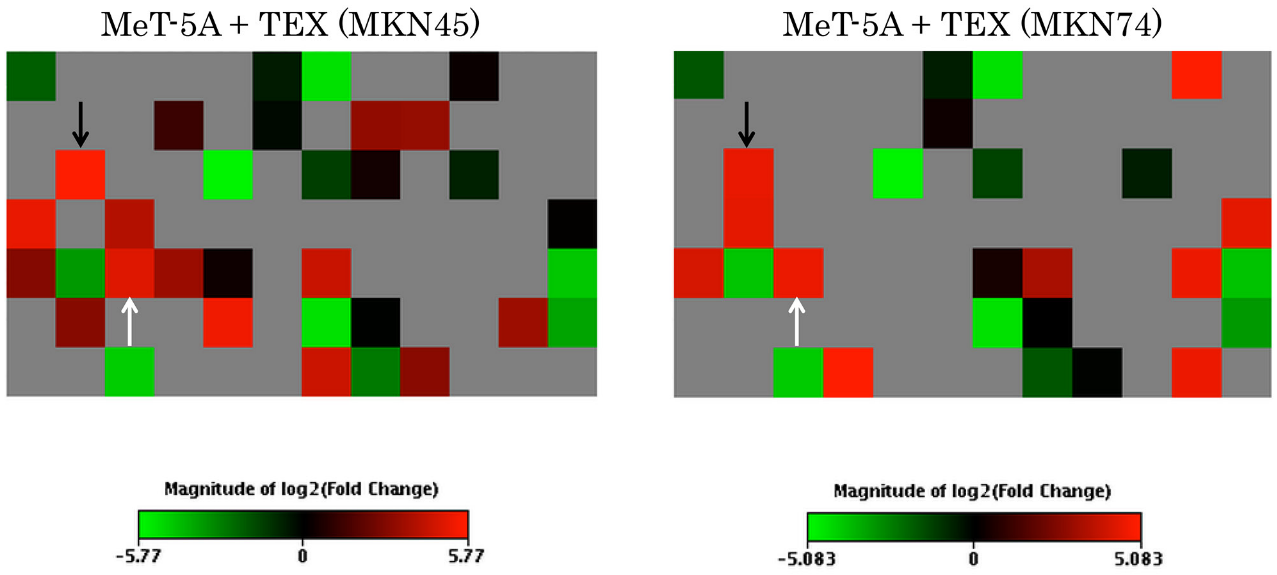

Supplementary Figure S2: The results of PCR array of ECM and adhesion-related gene targets. Black arrow; FN1, white arrow; LAMC1.

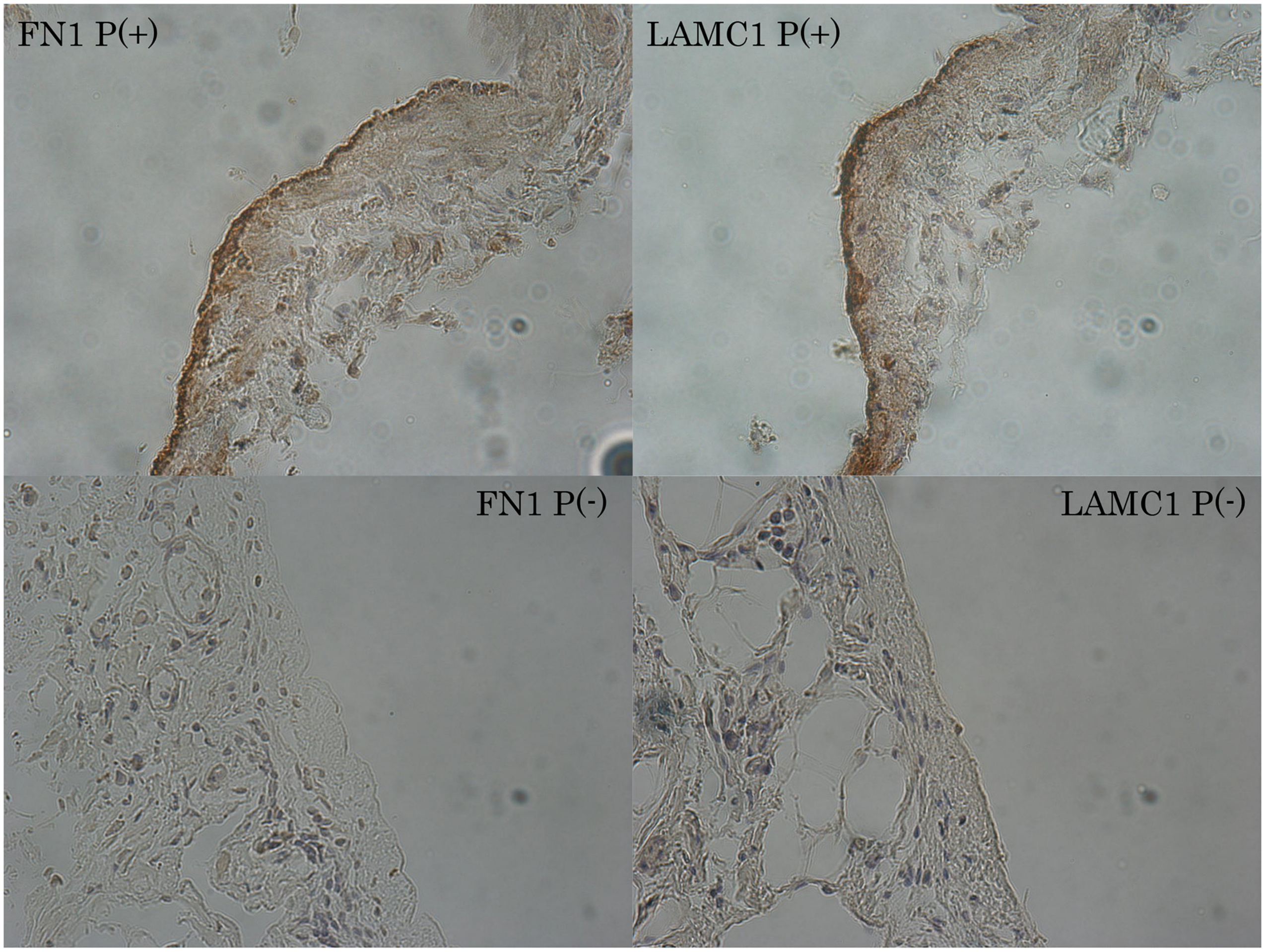

Supplementary Figure S3: Immunohistochemistry of peritoneal membrane.
